# Oblique lateral interbody fusion with internal fixations in the treatment for cross-segment degenerative lumbar spine disease (L2-3 and L4-5) finite element analysis

**DOI:** 10.1038/s41598-023-43399-x

**Published:** 2023-10-10

**Authors:** Shuyi Zhang, Yilong Zhang, Licai Huang, Shuao Zhang, Chenshui Lu, Zhengpeng Liu, Chan Kang, Zhao Wang

**Affiliations:** 1 Department of Orthopedics, Fuzhou Second Hospital, Fuzhou, 350007 Fujian China; 2 Department of Spine Surgery, Affiliated Hospital of Chengde Medical College, Chengde, 067000 Hebei China; 3 School of Civil Engineering, Lanzhou University of Technology, Lanzhou, 730000 Gansu China; 4 Department of Foreign Languages, Fu Zhou University, Fuzhou, 350100 Fujian China; 5 Department of Orthopedics, Chungnam National University Hospital, Daejeon, 35015 Republic of Korea

**Keywords:** Anatomy, Diseases, Medical research, Pathogenesis

## Abstract

Multi-segmental lumbar degenerative disease, including intersegmental disc degeneration, is found in clinical practice. Controversy still exists regarding the treatment for cross-segment degeneration. Oblique Lateral Interbody Fusion (OLIF) with several internal fixations was used to treat cross-segment lumbar degenerative disease. A whole lumbar spine model was extracted from CT images of the whole lumbar spine of patients with lumbar degeneration. The L2-3 and L4-5 intervertebral spaces were fused with OLIF using modeling software, the Pedicle screws were performed on L2-3 and L4-5, and different internal fixations were performed on L3-4 in Finite Element (FE) software. Among the six 10 Nm moments of different directions, the L3-4 no surgery (NS) group had the relatively largest Range of Motion (ROM) in the whole lumbar spine, while the L2-5 Long segmental fixation (LSF)group had the smallest ROM and the other groups had similar ROM. The ROM in the L1-2 and L5-S1 was relatively close in the six group models, and the articular cartilage stress and disc stress on the L1-2 and L5-S1 were relatively close. In contrast, the L3-4 ROM differed relatively greatly, with the LSF ROM the smallest and the NS ROM the largest, and the L3-4 Coflex (Coflex) group more active than the L3-4 Bacfuse (Bacfuse) group and the L3-4 translaminar facet screw fixation (TFSF) group. The stress on the articular cartilage and disc at L3-4 was relatively greater in the NS disc and articular cartilage, and greater in the Coflex group than in the Bacfuse and TFSF groups, with the greatest stress on the internal fixation in the TFSF group, followed by the Coflex group, and relatively similar stress in the Bacfuse, LSF, and NS groups. In the TFSF group, the stress on the internal fixation was greater than the yield strength among different directional moments of 10 Nm, which means it is unsuitable to be an internal fixation. The LSF group had the greatest overall ROM, which may lead to postoperative low back discomfort. The NS group has the greatest overall ROM, but its increased stress on the L3-4 disc and articular cartilage may lead to accelerated degeneration of the L3-4 disc and articular cartilage. The Coflex and Bacfuse groups had a reduced L3-4 ROM but a greater stress on disc compared to the LSF group, which may lead to disc degeneration in the long term. However, their stress on the articular cartilage was relatively low. Coflex and Bacfuse can still be considered better surgical options.

## Introduction

Lumbar interbody fusion (LIF) is a classic and effective surgical method of the treatment for degenerative lumbar spine disorders^1–5^. The method includes Anterior LIF (ALIF), Posterior LIF (PLIF), Transforaminal LIF (TLIF), Direct Lateral Interbody Fusion/Extreme Lateral Interbody Fusion (DLIF/XLIF), and Oblique Lateral Interbody Fusion (OLIF), etc. With the wide application of minimal invasion surgery, in 1997 Mayer^6^ first described a minimally invasive anterior approach to the lumbar spine through retroperitoneal access at the L2-L5 level and transperitoneal access at the L5-S1 level. In 2012, Silvestre et al.^7^ used a minimally invasive retroperitoneal anterior approach similar to Mayer’s approach for anterior lumbar interbody fusion. This technique, an aorta-psoas approach, is referred to by Silvestre et al. as OLIF. OLIF is gradually being accepted by many spine surgeons as the minimally invasive concepts are developed^8–10^. OLIF is performed mainly through the retroperitoneal abdominal vascular sheath and the physiological gap between the anterior border of the psoas major muscle to reach the diseased disc without opening the spinal canal and damaging the posterior muscles, ligaments, and bony structures^8^.

OLIF can allow for the tightening and retraction of the annulus fibrosus, posterior longitudinal ligament, and ligamentum flavum through effective gap opening and repositioning of the slipped vertebral body. It also can restore spinal stability through intervertebral fusion, thereby restoring the height of the intervertebral space, expanding the volume of the spinal canal, enhancing coronal balance, and eliminating the symptoms of spinal stenosis caused by dynamic compression^11–14^.

OLIF is primarily used for degenerative lumbar spine disease and can be used for single-level discogenic back pain to multi-level degenerative scoliosis^15^. However, controversy still surrounds OLIF, including whether internal fixation is still required for OLIF with CAGE.

Many patients with long-segment degenerative or cross-segment degenerative lumbar spine disease are prone to increased postoperative adjacent spondylolisthesis^16,17^. In a study of 37 single-segment fusion cases, Gillet reported potential adjacent motion segmental change in 32% of patients^18^. LIF with rigid spinal fixation reduced the ROM at the surgical level, which may result in increased motion at the adjacent level and greater disc stress^17^. The increased disc stress is thought to accelerate degeneration and in some case lead to adjacent segment degeneration^17,19–21^. After a long-term follow-up of patients with lower lumbar fusion, Lehmann et al. reported segmental instability above the fusion level in 45% of patients^22^. Fusion length has been confirmed as one of the most influential factors that causes ASD. Chen et al. reported that an increase in the number of fusion levels produced greater stress on the disc adjacent to the fusion level^17^.

The disc or segment that causes discomfort such as low back pain is named as the responsible disc or responsible segment. Many current studies showed that non-responsible discs, treated or untreated, did not make a significant difference in the long-term surgical outcome^23,24^. However, in our daily life, many intervals of the disc without symptoms exist. Current controversy exists regarding the segment of internal fixation in many patients present with non-adjacent two-segment degenerative disease in the lumbar spine. For example, when L2-3 and L4-5 are the responsible discs and L3-4 is the non-degenerative disc or is a non-responsible disc, there are various options for surgical approaches. Such as internal fixation is fixed at L2-5; Internal fixation is fixed at L2-3 and L4-5; Bacfuse is only fixed at L3-4; Coflex is only fixed on L3-4; translaminar facet screw is only fixed at L3-4.

Finite element analysis is used to analyze the biomechanical changes in the total lumbar spine after treatment of degenerative disease at L2-3 and L4-5 by OLIF with different internal fixations. The mechanical analysis of the different internal fixations is used to compare the non-responsible discs located in the two responsible discs, thus determining the more mechanically effective internal fixation method.

## Materials and methods

### Grouping (Fig. 1)

**Figure 1:**
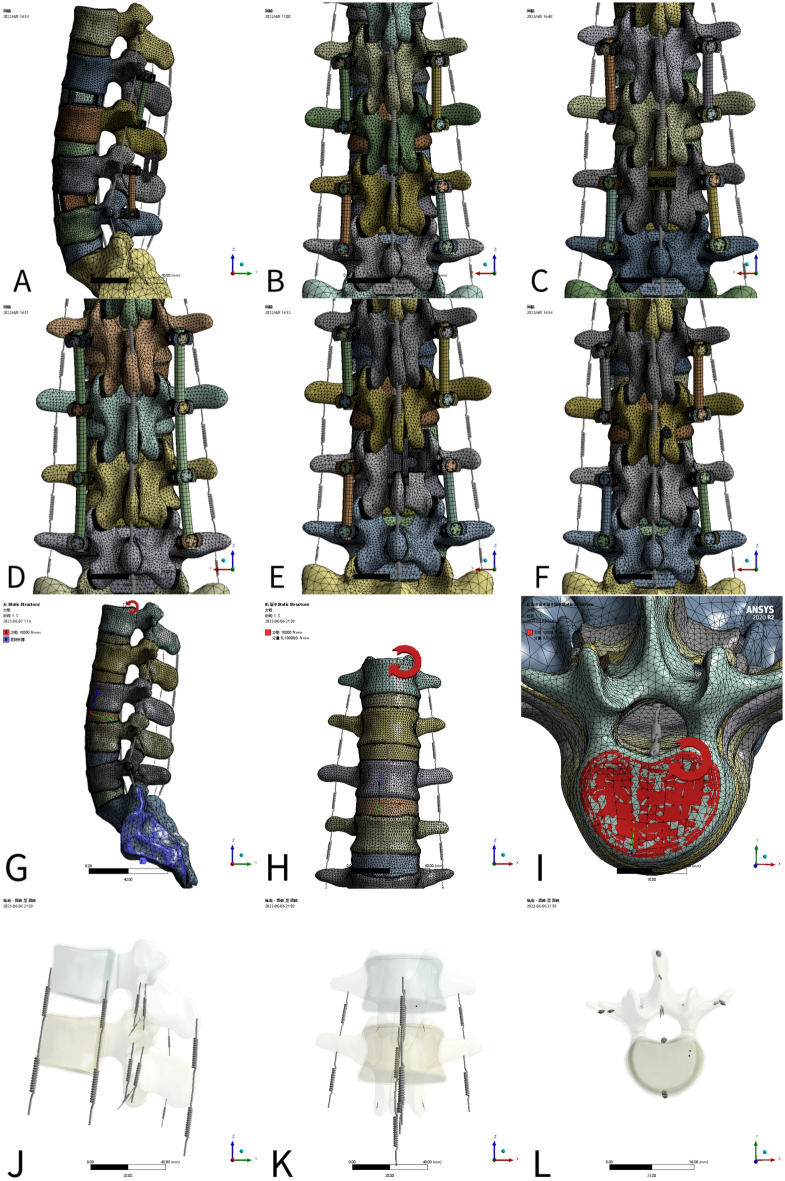
Model mesh division, boundary condition setting and ligament Composition (**A**) Bacfuse (lateral position); (**B**) NS; (**C**) Coflex; (**D**) LSF; (**E**) Bacfuse; (**F**) TFSF; (**G**) an intact model (flexion); (**H**) an intact model (right lateral bending); (**I**) an intact model (Right rotation); (**J**) L1-3 ligament Composition (sagittalsection); (**K**) L1-3 ligament composition (coronal position); (**L**) L1-3 ligament composition (horizontal position).

Six finite element models of the lumbar spine were created. The finite element models included (1) an intact model, (2) the L3-4 no surgery model (the NS model), (3) the L3-4 Coflex model (Coflex model), (4) the L2-5 Long segmental fixation model (the LSF model) (5) the L3-4 Bacfuse model (the Bacfuse model) (6) L3-4 translaminar facet screw fixation model (the TFSF model).

### Construction of the intact model

A volunteer with lumbar degenerative disease was selected. A total of 481 CT images (Siemens 128 slice 64-row, SOMATOM Definition AS spiral CT, Germany) with a slice thickness of 0.625 mm were provided by the Affiliated Hospital of Chengde Medical College. The CT images were stored in Digital Imaging and Communications in Medicine format (DICOM). The collected raw DICM data were imported into Mimics Research 21.0 (Materialise, Belgium) for three-dimensional (3D) reconstruction. Subsequently, the 3D model generated by Mimics was imported into Geomagic wrap 18 (reverse engineering software, USA); The noise and smoothing model were removed and cancellous bone and posterior structure were created and imported into SolidWorks 2020 (CAD software, Dassault Systemes, USA). Articular cartilage and intervertebral disc were created and the nucleus pulposus accounted for approximately 50% of the discs^25–30^. The thickness of the cortical bone was 1 mm and the thickness of the vertebral endplate and cartilage endplate was set as 0.5 mm^25–30^. The models with internal fixations were created in this software and were assembled with the lumbar spine model. The L2-3 and L4-5 discs of the patient's lumbar spine model were processed, including the removal of the cartilage endplates, fibrous rings, and all of the nucleus pulposus which are required to be removed in the OLIF, and CAGE was inserted into the L2-3 and L4-5 intervertebral spaces^8,31^, pedicle screws were inserted into L2-L3 and L4-L5^32^. The LSF group used two titanium rods to fix L2-5. The NS group used four titanium rods to fix the L2-3 and L4-5 vertebrae. Bacfuse^33^ was fixed on L3-4; Coflex^34^ was fixed on L3-4. The TFSF group used translaminar screws from the right side of the L3 through to the left superior articular process of L4^35^.

### Meshing and boundary condition setting (Fig. 1)

These models were imported into ANSYS Workbench 2020 R2 (ANSYS, Ltd., Canonsburg, Pennsylvania, USA) for preprocessing, and corresponding material parameters were set for each component (Table 1)^26,36–38^. Ligaments were simulated using springs subject only to pullout force (Table 1)^39–42^.

**Table 1:** Material properties and ligament property of each part of the FE (a for material properties; b for ligament properties).

Component/materials	Elastic modulus (MPA)	Poisson ratio		
Cortical bone	12,000	0.3		
Cancellous bone	100	0.2		
Posterior structures	3500	0.25		
Anulus fibrosus	4.2	0.45		
Vertebral endplate	12,000	0.3		
Cartilage endplate	25	0.4		
Articular cartilage	50	0.3		
Titanium alloy	110,000	0.3		
Cage (polyetheretherketone, peek)	3600	0.3		
Allogeneic bone	3500	0.3		
Nucleus pulposus	1	0.499		

Component/ligaments	Elastic modulus (MPA)	A (MM^2^)	L (mm)	K = (A.E)/L (kg·M-2·S-2)
Anterior longitudinal ligament (ALL)	7.8	22.4	20	8.74
Supraspinal ligament (SSL)	8	10.5	22	3.82
Posterior longitudinal ligament (PLL)	10	7	12	5.83
Intertransverse ligament (ITL)	10	0.6	32	0.19
Capsular ligament (CL)	7.5	10.5	5	15.75
Interspinal ligaments (ISL)	10	14.1	13	10.85
ligamentum flavum (LF)	17	14.1	15	15.98

The number of nodes and elements in the model is shown in the Table 2 and Fig. 1 The contact type of facet joint was friction with a friction coefficient of 0.1^25^. The contact type of the Interspinous Process device and upper and spinous process was friction with friction coefficient 0.8^43^. The remaining contact types were set to the binding mode. To improve the efficiency and accuracy of the calculation, the type was set to a tetrahedral elastic element^44,45^, the size of the articular cartilage mesh was 0.5 mm, the size of screw and bolt was 1 mm, and the rest of the objects were set to 2 mm.

**Table 2:** Number of nodes and elements after grid subdivision for various models.

Model node element	Model node element	Model node element
Intact model	1,082,515	730,659
NS model	1,565,232	1,004,590
Coflex model	1,594,910	1,021,483
LSF model	1,564,431	1,004,347
Bacfuse model	1,606,580	1,029,488
TFSF model	1,592,281	1,018,759

Six models were set up with boundary and loading conditions in the Static model^26,37^: bilateral alar sacralis fixation in S1, a vertical axial downwards preload of 150 N applied to the upper surface of L1 (150 N represents the weight of the patient’s upper body^46^), and a 10 Nm moment along the radial direction on the upper surface of L1 to simulate six different physiological motions. These motions were: flexion, extension, right and left bending, and right and left axial rotation. The biomechanical stability of OLIF with different fixations was investigated by analyzing and comparing ROM, CAGE, and internal fixation stress.

### Ethics approval and consent to participate

The present study was approved by the Ethics Committee of the Affiliated Hospital of Chengde Medical College. Informed consent obtained from each participant was written. All protocols are carried out in accordance with relevant guidelines and regulations.

### Consent for publication

Informed consent obtained from each participant was written. All protocols are carried out in accordance with relevant guidelines and regulations.

## Results

### Mesh convergence test (Table 3) and validation of the model (Fig. 2)

**Table 3:** Mesh convergence test.

	Mesh 1.5	Mesh 2	Mesh 2.5	Mesh 3
Node number	1,903,844	1,082,515	789,216	644,458
Element number	1,310,968	730,659	525,161	425,407
Cortical bone max stress (Mpa)	11.258	10.868	12.038	8.7518
Rate of change compared with mesh 0.5 mm		3.46%	6.92%	22.26%
Endplate max stress (Mpa)	14.685	14.371	13.292	13.207
Rate of change compared with mesh 0.5 mm		2.14%	9.49%	10.06%
Nucleus max stress (Mpa)	0.17341	0.17217	0.17779	0.15358
Rate of change compared with mesh 0.5 mm		0.71%	0.43%	11.43%
Fiber max stress (Mpa)	0.82743	0.827939	0.78812	0.77454
Rate of change compared with mesh 0.5 mm		0.06%	4.75%	6.39%

**Figure 2:**
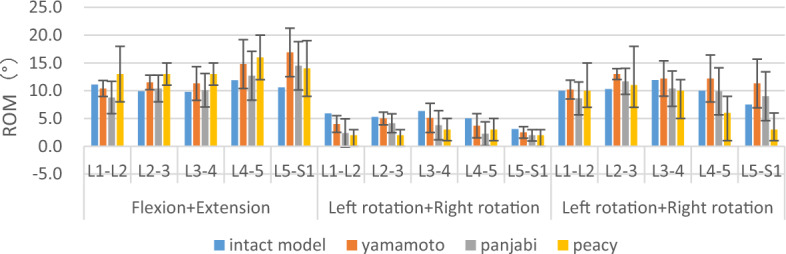
The comparison of the ROM between the intact model and the previous in vitro experimental study. Note: The bars indicated standard deviation in the experiment or the range of motion given in the text.

Different mesh sizes may have different results. Though the smaller mesh increases the operation time, the result is relatively reliable. To find a suitable mesh size for this test, the mesh size of cortical bone and cancellous bone, endplate, annulus fibrosus and nucleus pulposus of L1-5 vertebral body in the intact model was made into Mesh 1.5 mm, Mesh 2 mm, Mesh 2.5 mm and Mesh 3 mm. Each group was subjected to a moment of 150 N perpendicular to the L1 vertebral body; The percentage difference of Von-Mises stress between Mesh 1.5 mm score and Mesh 2 mm, Mesh 2.5 mm and Mesh 3 mm is shown in Table 3. The von Mises stress difference between Mesh 1.5 mm and Mesh 2 mm is less than 5% in most tissues. Therefore, Mesh 2 mm is considered to be convergent, which can not only ensure the simulation results, but also improve the efficiency^40,45^.

After applying similar loads to the models, the ROM results with those of previous in vitro experiments were compared^47–50^. The results were found to be consistent with those of the reported data. All segmental ROM was within the effective range or average standard deviation of the cadaveric experiments.

### Overall ROM of different groups (Table 4)

**Table 4:** Ranges of motion predicted by the intact FE model compared with reported ROMs from in vitro study.

	Intact model (%)	L3-4 NS (%)	L3-4 coflex (%)	L3-4 LSF (%)	L3-4 bacfuse (%)	L3-4 TFSF (%)
Flexion	100.00	62.40	50.59	46.79	47.67	50.07
Extension	100.00	58.76	41.08	39.23	39.92	41.82
Left bending	100.00	63.06	58.90	38.05	46.94	51.87
Right bending	100.00	61.75	57.40	36.49	45.36	48.23
Left rotation	100.00	61.95	57.78	43.79	50.97	48.63
Right rotation	100.00	59.88	54.94	41.50	48.53	54.08

Under the same loadings, we compared the ROM of different groups with that of the intact model. The NS group had the greatest overall ROM under all loading conditions, and the LSF group had the smallest overall ROM under all loading conditions. The Bacfuse group had similar overall ROM with the LSF group in flexion and extension, but greater ROM in lateral bending and rotation than the LSF group. Among the models with internal fixation in L3-4, the Coflex group had the greatest ROM. The TFSF group had a similar ROM to the Coflex group in flexion and extension. The TFSF group had less ROM than the Coflex group in right and left lateral bending. The TFSF group had more ROM than the Bacfuse and LSF groups in rotation, and similar ROM to the Bacfuse group in rotation, but slightly more ROM than the Bacfuse group.

### Relative ROM of L1-2 and L5-S1 (Table 5)

**Table 5:** ROM of L1-2 to L5-S.

	Exercise load	Intact model (%)	L3-4 NS (%)	L3-4 coflex (%)	L3-4 LSF (%)	L3-4 bacfuse (%)	L3-4 TFSF (%)
L1-2	Flexion	100	101.13	101.92	101.34	101.39	101.39
	Extension	100	99.41	100.80	99.91	99.85	99.67
	Left bending	100	102.91	103.89	102.89	103.07	103.07
	Right bending	100	100.85	101.67	100.83	100.80	100.78
	Left rotation	100	101.16	102.45	101.11	101.04	101.03
	Right rotation	100	100.87	102.20	100.91	100.88	100.87
L5-S1	Flexion	100	102.50	102.79	102.63	102.52	102.60
	Extension	100	101.23	103.12	101.79	101.68	101.22
	Left bending	100	102.06	102.33	102.47	102.33	102.36
	Right bending	100	100.38	100.82	100.95	100.93	100.77
	Left rotation	100	101.99	102.61	102.21	102.30	102.33
	Right rotation	100	99.52	100.52	99.75	99.90	99.71

The relative ROM of the L1-2 and L5-S1 segments for the different models was close under different directions of 10 Nm loading.

### ROM of interverbral disc (L3-4) (Fig. 3)

**Figure 3:**
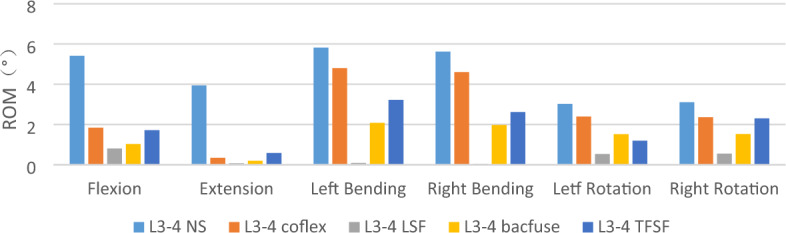
ROM of interverbral disc (L3-4).

In terms of the ROM of L3-4 in the surgical models, the LSF group had the relatively smallest ROM and the NS group had the relatively greatest ROM. The Bacfuse group had relatively good ROM restriction of the L3-4 gap, and its L3-4 ROM was only greater than the LSF group under different loadings, but the ROM of the TFSF group was less than that of the Bacfuse group in left rotation, while the ROM of the TFSF group was greater than that of the Bacfuse group in other physiological motions. Only in extension, the ROM of the Coflex group was greater than that of the NS group. In other physiological motions, the ROM of the Coflex group was smaller than that of the NS group.

### Von Mises stress of Intervertebral disc (L3-4) (Figs. 4 and 5)

**Figure 4:**
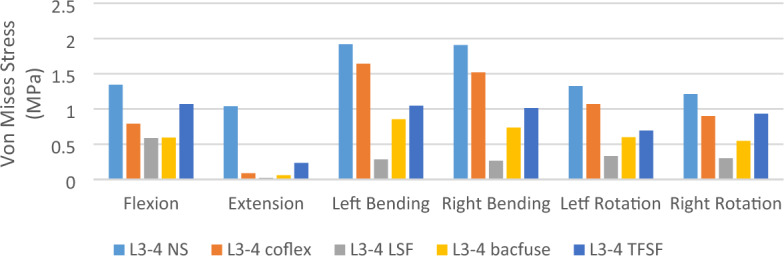
Von Mises stress of Intervertebral disc (L3-4).

**Figure 5:**
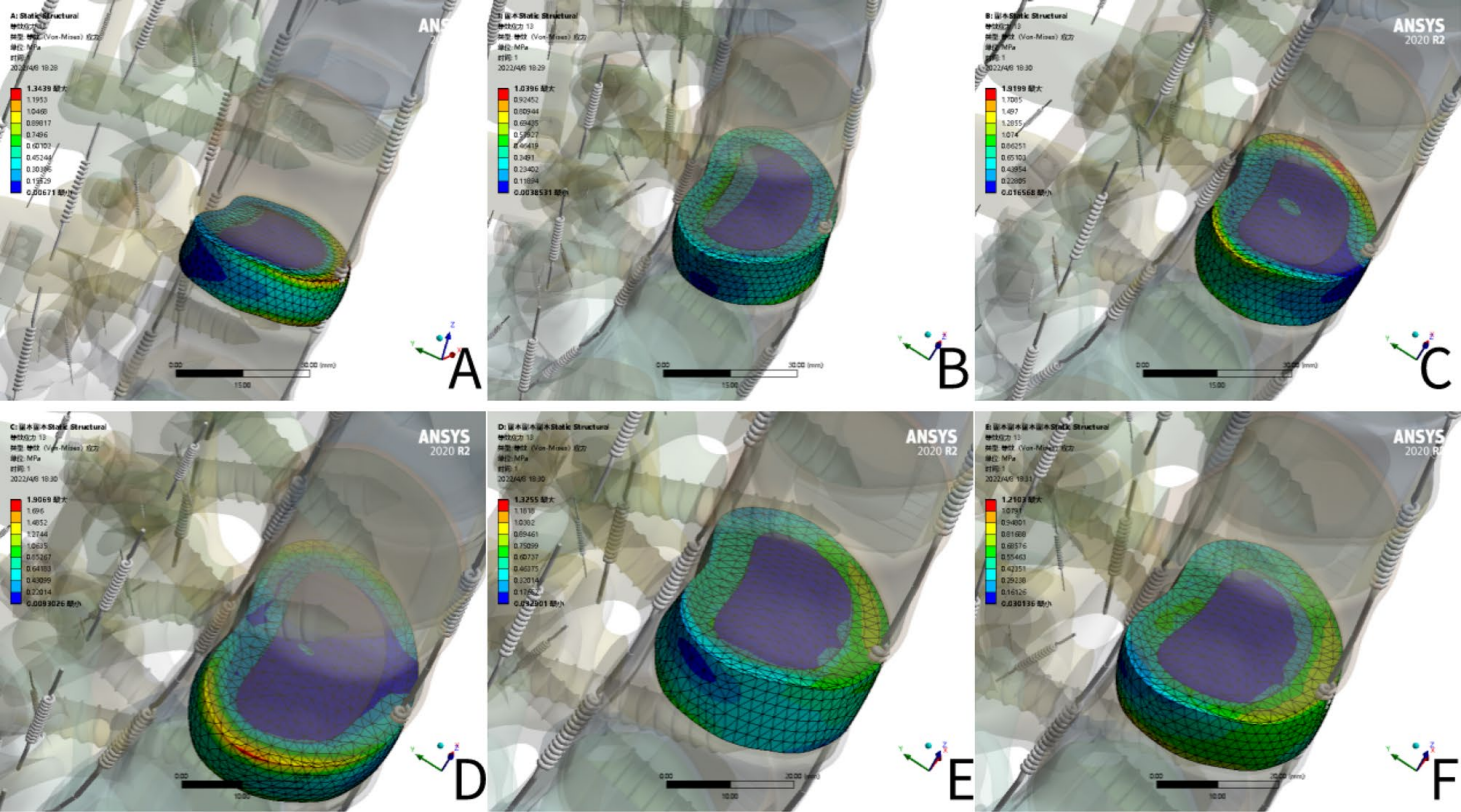
Von Mises stress cloud of NS group L3-4 Intervertebral disc (**A** flexion, **B** extension, **C** left bending, **D** right bending, **E** right rotation, **F** left rotation).

The stress on the L3-4 articular cartilage of the NS group was the highest and the stress on the LSF group was the lowest under different loading conditions. In bending and rotation, the stress on the L3-4 disc in the Bacfuse group was significantly greater than that in the LSF group but less than that in the other groups.

### L3-4 Von Mises stress of articular cartilage (L3-4) (Fig. 6)

**Figure 6:**
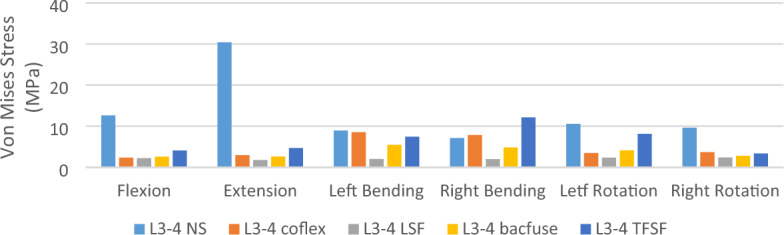
Von Mises stress of articular cartilage (L3-4).

The stress on the articular cartilage of L3-4 in the NS group was less than that in the other groups under various loads. The stress on the articular cartilage of L3-4 in the NS group was biggest in flexion, extension, and axial rotation, while in left and right bending, the stress on the articular cartilage of the NS group was relatively similar to that of the Coflex group, but in left bending the stress on the articular cartilage of the TFSF group was greater than that of the other models, which may be caused by the direction of TFSF passing through the intervertebral disc and screw passing through the articular cartilage.

### Von Mises stress on L3-4 internal fixation (Figs. 7 and 8)

**Figure 7:**
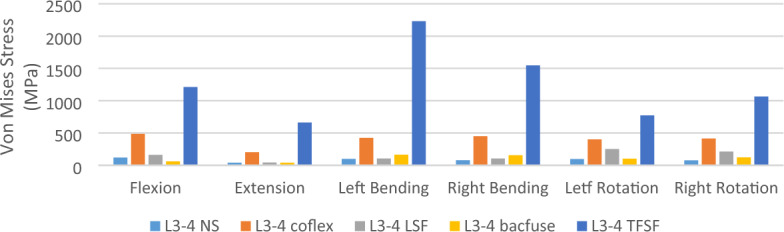
Von Mises stress of internal fixation.

**Figure 8:**
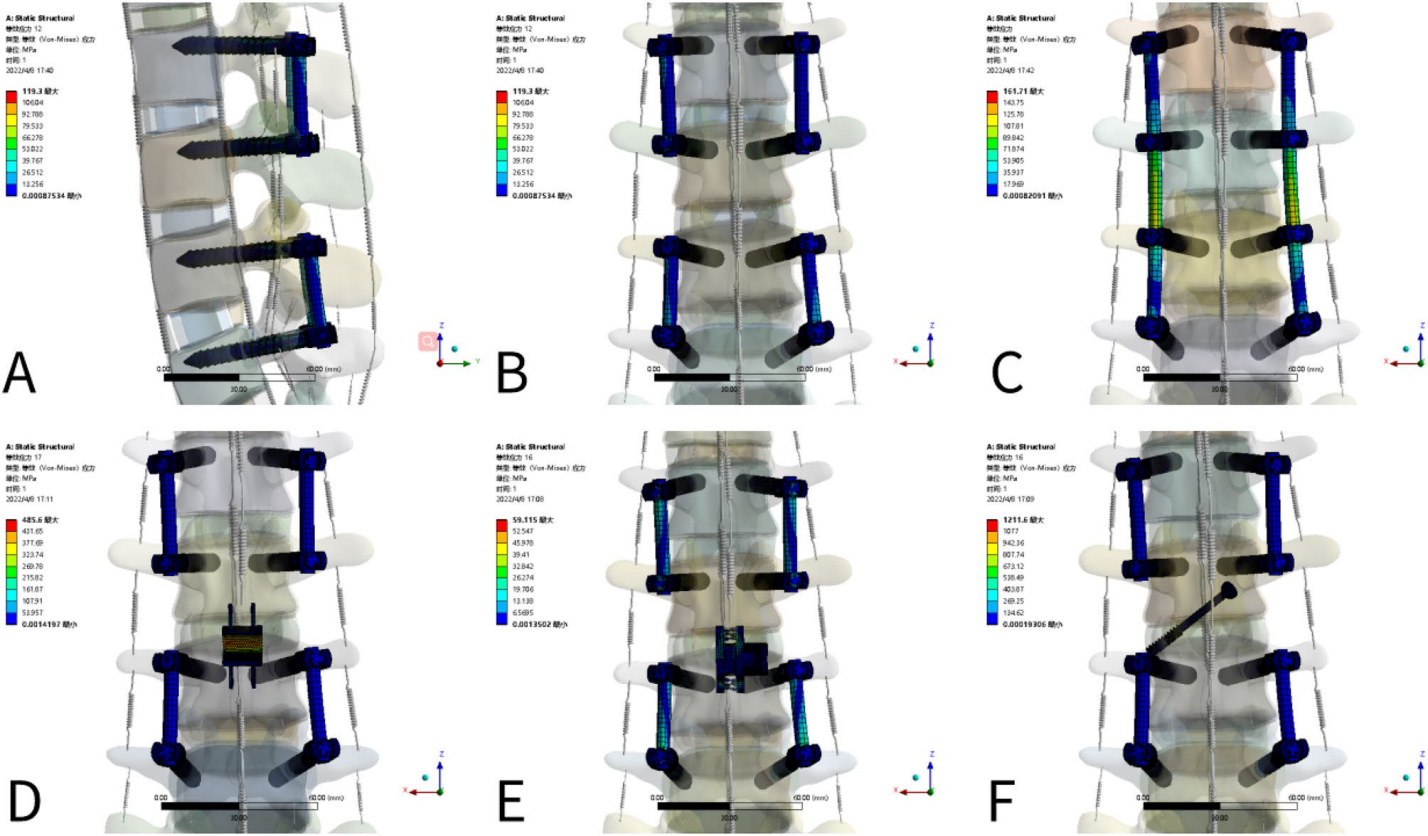
Von Mises stress of internal fixation in flexion in each group [**A**, NS (sagittal plane), **B**, NS (coronal position), **C**, LSF (coronal position), **D**, Coflex (coronal position), **E**, Bacfuse (coronal position), **F**, TFSF (coronal position)].

The stress in the TFSF group was greater than that in other groups, while the stress in the Coflex group was less than that in the TFSF group but still greater than that in the other internal fixations. In extension, the stress in the NS group was close to that in the Bacfuse and TFSF groups. In flexion, the stress in the NS group was close to that in the TFSF group and greater than the stress in the Bacfuse group. In left and right lateral bending, the stress in the BacFuse group was greater than that in the LSF and NS groups; the stress in the LSF and NS groups was close. In left and right rotation, the stress in the Bacfuse group was slightly greater than that in the LSF and NS groups.; the stress in the NS group was smallest.

### L1-2 and L5-S1 intervertebral disc Von Mises stress and articular cartilage Von Mises stress (Figs.9, 10, 11, 12)

**Figure 9:**
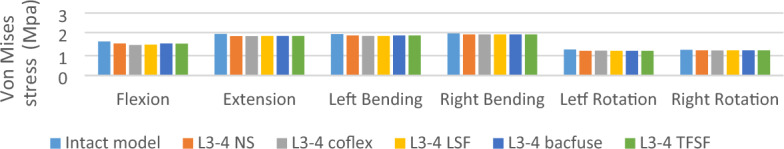
Von Mises stress of intervertebral disc (L1-2).

**Figure 10:**
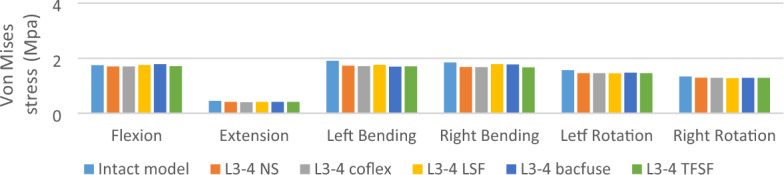
Von Mises stress of intervertebral disc (L5-S1).

**Figure 11:**
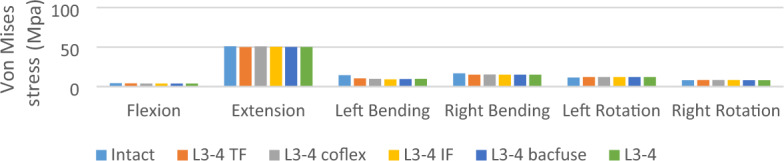
Von Mises stress of articular cartilage (L1-2).

**Figure 12:**
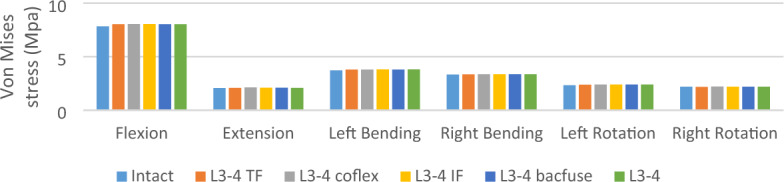
Von Mises stress of articular cartilage (L5-S1).

Under different loadings, the implantation of different internal fixations has litte effects on the stress of the disc and articular cartilage of L1-2 and L5-S1.

## Discussion

OLIF, one of the most popular minimally invasive lumbar fusion procedures, can better reduce complications associated with surgical approach and preserve posterior column stability^51,52^. However, due to current instrumentation limitations, direct decompression is not possible^15,53^. Thanks to the CAGE which can hold up the intervertebral space well and provide indirect decompression, OLIF can restore intervertebral foramen height, tense the posterior longitudinal ligament and restore the sagittal sequence of the spine^8,54^. Currently, there is a risk of subsidence following fusion implantation in OLIF alone. Therefore, more and more spine surgeons are performing OLIF with internal fixation to avoid CAGE subsidence and improve the stability of the operated segment^55^. OLIF with internal fixation also reduces fusion loosening and improves intervertebral fusion^56–59^. Pedicle screw is the gold standard for internal fixation in spine surgery as it maintains the stability of the three columns of the vertebral body^60^.

Currently, finite element analysis is mainly used in lumbar spine disease to analyze the mechanics of a single segment after surgery^10^. However, as the elderly population and the number of patients with degenerative lumbar disease increase, there is a large number of multi-segmental degeneration, including inter-segmental degeneration. As one to two segments in the middle of the intersegmental segment are asymptomatic or without surgical indications, discs without surgical indications are generally not treated to reduce the trauma and cost of surgery. But the untreated intersegmental discs are the adjacent vertebrae of the surgical segment on either side, which may change the mechanical properties of the adjacent vertebrae after surgery. The changes may result in discs and articular cartilage degeneration, which may produce corresponding nerve roots as adjacent segment degeneration worsens^61^. In a serious case, a second surgery is needed. OLIF is commonly performed on the L2-L5^8^. In this study, the L2-3 and L4-5 were treated with OLIF, pedicle screws, and titanium rods. We discussed and analyzed whether to use a long titanium rod to fix L2-L5 or use Coflex, Bacfuse, or translaminar facet screw to fix L3-4. The ROM, disc, and articular cartilage stress on the L1-2 and L5-S1 was relatively similar. Although under different loadings, the model with internal fixations may have greater ROM at L1-2 and L5-S1 than the intact model. That may be because the internal fixations will limit the movement of the lumbar spine; L1-2 and L5-S1 are active segments, which may compensate for the decrease in the movement caused by internal fixations at L2-5.The different internal methods have little effect on the disc and articular cartilage degeneration of L1-2 and L5-S1. The results for the intermediate segment (L3-4) were different and the effects of different internal fixation on the intermediate segment (L3-4) and the whole lumbar spine were discussed below.

### The NS group and the LSF group

10 Nm moment was applied in the NS group from different directions. To reduce the ROM decrease in lumbar spine after surgery, L3-4 was not fixed. It was found that the ROM of the L3-4 was greater than that of the other surgical models, resulting in increased stress on the L3-4 intervertebral disc and L3-4 articular cartilage. According to the "Wolff" law^62^, stress change on the articular cartilage leads to accelerated degeneration of the facet joint. If the degeneration is severe, related symptoms may occur. The increase in the relative ROM of the disc and stress on the disc can lead to disc degeneration^63^.

The LSF group had the lowest overall ROM in this study, with an overall RON equivalent to 40% of the intact model. If a patient wants to achieve the pre-operative ROM, he needs to make more effort. In this group, the stress on the cartilage and disc of L3-4 was the smallest, and the relative ROM of L3-4 was also the smallest, with a lower likelihood of postoperative degeneration in L3-4^63^. In terms of overall internal fixation stress, the NS group had the lowest overall stress and the lowest risk of screw and rod breakage compared to the LSF group.

### The Coflex and Bacfuse groups

Dynamic lumbar spine fixation techniques are gradually used in the treatment for degenerative lumbar spine diseases such as degenerative lumbar spinal stenosis, discogenic lower back pain, synovial syndrome, disc herniation, and mild lumbar instability as research into the physiological function of the spine and biomechanics continues, and the functional spinal unit (FSU) has been established^64^. The interspinous process system (ISPS) has been widely used as a non-fusion procedure and has achieved good results. The procedure can open the spinous process gap and limit the extension of the corresponding segment to a certain extent, thereby increasing the spinal canal and intervertebral foramen and reducing the stress on the posterior facet joints and anterior foramina^65^. The overall lumbar ROM in the Coflex group was relatively good. The ROM in the Bacfuse group was only less than of the LSF group. The stress on the L3-4 articular cartilage and disc in the Coflex group was relatively great. The stress on the L3-4 articular cartilage and disc in the Bacfuse group was relatively small but greater than that in the LSF group. The purpose of using the interspinous process device was to reduce the excessive ROM of L3-4 after L2-3 and L4-5 fusion, and the degeneration caused by the increased stress on the interspinous disc and articular cartilage. Cunningham et al.^66^ studied spinal instability and intradiscal pressure and found that increased stress on the disc altered the metabolic production and exchange of disc material and that the increase in the stress on the disc mobility would lead to disc deformation and accelerate disc degeneration. In a biomechanical study of lumbosacral fusion, Lee et al.^67^ found that rigid fixation resulted in excessive movement of the adjacent segment, which would lead to adjacent segment degeneration.

### TFSF group

In 1984, Magerl^35^ first reported the technique of translaminar facet screw. The screws were inserted from the right L3 lamina of vertebra through the left articular cartilage and into the superior L4 articular process to reduce ROM and the stress on articular cartilage and intervertebral disc. The stress on the internal fixation of the articular process screw was too high, exceeding the yield strength of the titanium of 897–1034 Mpa^68^, which may result in a risk of nail breakage. However, according to stress cloud analysis, we found that the stress was mainly concentrated on the titanium rod, especially between the superior and inferior articular processes of L3-4.

The TFSF group was similar to the Bacfuse group in terms of the ROM restriction, and its stress on the articular cartilage was relatively high, which is only less than the NS group under many loadings. The stress on the L3-4 disc was also relatively higher in the TFSF group. Therefore, TFSF is considered the last option.

### Limitations

In this experiment, a simplified model was used and the influence of muscle, skin, and viscera was ignored. The model used in this experiment is a skeletal model extracted from the volunteer's CT scans, which has been optimised by the relevant software for smoothness and so on. There are experimental errors. There are no related clinical studies and cadaveric experiments with our experiment.

## Conclusion

Biomechanical or clinical controversy remains as to whether to fix the intermediate segment (L3-4), which is without a surgical pointer, while using a titanium rod to directly fix L2-5 or to fix the L3-4 segment alone with other elastic fixations. OLIF with the Pedicle screw fixations was performed in L2-3 and L4-5. Different internal fixations were used in L3-4 and grouped accordingly. It was found that ROM in L1-2 and L5-S1 as well as articular cartilage and disc stress were less affected. by the simulation software at a moment of 10 Nm, with changes in ROM mainly in L3-4. The L3-4 ROM of the NS group may even be greater, which may accelerate disc degeneration, thus requiring reoperation later. If the risk of disc degeneration is greater, L3-4 are fixed simultaneously. The NS group still maintained its ROM. In the LSF group, the ROM was greatly limited. To achieve the same pre-operative overall ROM, more efforts are required, which may result in reduced lumbar mobility after surgery. The Coflex and Bacfuse groups had better overall ROM than the LSF group, but they also experienced disc degeneration and articular cartilage degeneration in the long term. Coflex and Bacfuse can be an alternative to LSF, and may also be a better alternative in some cases. The TFSF group has greater stress than the yield strength of the internal fixation because of the 10Nm moment in different directions, which results in a greater risk of nail breakage. Therefore, TFSF is not a suitable alternative.However, a comprehensive analysis of relevant clinical trials is still needed.

## Data Availability

The datasets used and/or analyzed during the current study are available from the corresponding author on reasonable request. Readers can access the data and material supporting the conclusions of the study by contacting Shuyi Zhang at 915368073@qq.com.

## Abbreviations

OLIF: Oblique lumbar interbody fusion
FEA: Finite element analysis
CT: Computed tomography
Dicom: Digital imaging and communications in medicine format
ROM: Range of motion
ASD: Adjacent segment degeneration
PLIF: Posterior lumbar interbody fusion
TILF: Transforaminal lumbar interbody fusion
ALIF: Anterior lumbar interbody fusion
DLIF/XLIF: Direct lateral interbody fusion/ extreme lateral interbody fusion
NS model: L3-4 no surgery model
coflex model: L3-4 coflex model
LSF model: L2-5 Long segmental fixation model
Bacfuse model: L3-4 Bacfuse model
TFSF model: L3-4 translaminar facet screw fixation model
FSU: Functional spinal unit
ISPS: Interspinous process system
