# Author Correction: Oblique lateral interbody fusion with internal fixations in the treatment for cross-segment degenerative lumbar spine disease (L2-3 and L4-5) finite element analysis

**DOI:** 10.1038/s41598-023-46704-w

**Published:** 2023-11-08

**Authors:** Shuyi Zhang, Yilong Zhang, Licai Huang, Shuao Zhang, Chenshui Lu, Zhengpeng Liu, Chan Kang, Zhao Wang

**Affiliations:** 1 Department of Orthopedics, Fuzhou Second Hospital, Fuzhou, 350007 Fujian China; 2 Department of Spine Surgery, Affiliated Hospital of Chengde Medical College, Chengde, 067000 Hebei China; 3 School of Civil Engineering, Lanzhou University of Technology, Lanzhou, 730000 Gansu China; 4 Department of Foreign Languages, Fu Zhou University, Fuzhou, 350100 Fujian China; 5 Department of Orthopedics, Chungnam National University Hospital, Daejeon, 35015 Republic of Korea

Correction to: *Scientific Reports* https://doi.org/10.1038/s41598-023-43399-x, published online 10 October 2023

The Funding section in the original version of this Article was omitted. The Funding section now reads:

“Fujian Provincial Clinical Medical Research Center for First Aid and Rehabilitation in Orthopaedic Trauma (2020Y2014).”

The original Article has been corrected.

